# Obituary

**Published:** 1883-06

**Authors:** C. Diehl

**Affiliations:** Secretary Staff Buffalo General Hospital; Buffalo


### Obituary.

At a special meeting of the medical staff of the Buffalo General Hospital, convened May 23, 1883, on account of the death of Dr. John Gray, of Avon, Livingston county, N. Y., the following minute was directed to be spread upon the records:

"Dr. John Gray, senior assistant resident physician of the Buffalo General Hospital, died May 21, 1883, of scarlet fever of diptheretic character, after an illness of four days, contracted from a patient in the hospital, in the discharge of his duty.

"Dr. Gray was but twenty-three years of age, graduated in medicine at the medical department of the University of Buffalo in February, 1883, received the appointment of senior assistant resident physician, on competitive examination, and entered upon the discharge of his duties March 1, 1883.

"Short as was his residence in the hospital, he fulfilled the promise of his collegiate career. He was studious, diligent, attentive, intelligent, kind and courteous. There was in him the material for making a great and good physician, and so he would have builded himself. The hospital has sustained a great loss, the medical profession a greater, and his family a bereavement which may be imagined but cannot be expressed. To them the medical staff of the Buffalo General Hospital extend their most profound condolence and sympathy."

It was moved, seconded and carried that a copy of the above minute be published in the BUFFALO MEDICAL JOURNAL, and also that one be sent to the father of the deceased.

Respectfully yours,

C. DIEHL,

Secretary Staff Buffalo General Hospital.

BUFFALO, May 27, 1883.

